# Genome-wide characterization and expression analyses of the *auxin/indole-3-acetic acid* (*Aux/IAA*) gene family in barley (*Hordeum vulgare* L.)

**DOI:** 10.1038/s41598-020-66860-7

**Published:** 2020-06-24

**Authors:** Qi Shi, Yueya Zhang, Vinh-Trieu To, Jin Shi, Dabing Zhang, Wenguo Cai

**Affiliations:** 1 Key Laboratory of Ministry of Education for Conservation and Utilization of Special Biological Resources in Western China, Ningxia University, Yinchuan, Ningxia 750021 China; 2 College of Life Science, Ningxia University, Yinchuan, Ningxia 750021 China; 3 Joint International Research Laboratory of Metabolic and Developmental Sciences, State Key Laboratory of Hybrid Rice, School of Life Sciences and Biotechnology, Shanghai Jiao Tong University, Shanghai, 200240 China; 4 School of Agriculture, Food and Wine, University of Adelaide, Urrbrae, SA 5064 Australia; 5 Flow Station of Post-doctoral Scientific Research, School of Life Sciences and Biotechnology, Shanghai Jiao Tong University, Shanghai, 200240 China

**Keywords:** Plant breeding, Plant development, Plant domestication, Plant genetics, Plant signalling

## Abstract

*Aux/IAA* genes are early auxin-responsive genes and essential for auxin signaling transduction. There is little information about *Aux/IAAs* in the agriculturally important cereal, barley. Using *in silico* method, we identified and subsequently characterized 36 *Aux/IAAs* from the barley genome. Based on their genomic sequences and the phylogenic relationship with *Arabidopsis* and rice *Aux/IAA*, the 36 *HvIAAs* were categorized into two major groups and 14 subgroups. The indication of the presence or absence of these domains for the biological functions and acting mechanisms was discussed. The *cis*-element distributions in *HvIAA* promoters suggests that the *HvIAAs* expressions may not only regulated by auxin (the presence of AuxREs and TGA-element) but also by other hormones and developmental and environmental cues. We then studied the *HvIAAs* expression in response to NAA (1-Naphthaleneacetic acid) using quantitative real-time PCR (qRT-PCR). Like the promoter analysis, only 14 *HvIAAs* were upregulated by NAA over two-fold at 4 h. *HvIAAs* were clustered into three groups based on the spatiotemporal expression data. We confirmed by qRT-PCR that most *HvIAAs*, especially *HvIAA3, HvIAA7, HvIAA8, HvIAA18, HvIAA24* and *HvIAA34*, are expressed in the developing barley spike compared within seedling, suggesting their roles in regulating spike development. Taken together, our data provide a foundation for further revealing the biological function of these *HvIAAs*.

## Introduction

**Figure 1:**
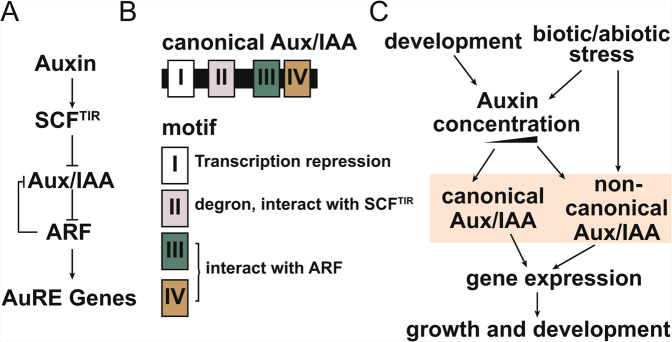
Aux/IAAs participate in the auxin signaling pathway. (**A**) The canonical auxin pathway. (**B**) The structure of canonical Aux/IAAs. (**C**) Aux/IAAs integrate the development and environment cues to modulate growth and development. SCF^TIR^: Auxin Transport Inhibitor Response 1- SKP1-Cullin-F-box complex; ARF: Auxin Response Factor; AuRE genes: Auxin response gene; I, II, III and IV: the conserved domain of Aux/IAAs.

Auxin plays a vital regulatory role in plant growth and development processes. *Aux/IAAs* belong to primary/early auxin-response genes including *GH3 (Gretchen Hagen 3)* and *SAUR (small auxin up RNA)*^1,2^. The transcription of these genes responds to auxin treatment quickly and they play an important role at the early stage in auxin signal transduction. Aux/IAAs inhibit the function of the transcription factors ARFs (auxin response factor) by the physical interaction with ARFs^3^. The SCF^TIR1^ protein complex (Auxin Transport Inhibitor Response 1-SKP1-Cullin-F-box complex) can sense auxin and degrade the Aux/IAAs expression level through auxin concentration dependent ubiquitin-mediated pathway^4^. Therefore, the Aux/IAAs mediate the release of ARFs with auxin level to activate auxin response gene expression (Fig. 1A)^5^.

The canonical Aux/IAA proteins contain four highly conserved domains (domains I-IV), which underlie the functional properties of these proteins (Fig. 1B)^6^. The domain I of N-terminal acts as a transcriptional repressor, it has an epistatic effect on the transcriptional activation of ARF^7^. Domain II has a specific sequence containing 13 amino acids, forming a degron to regulate the stability of Aux/IAA protein through interaction with ubiquitination complex TIR1^8,9^. The half-life of Aux/IAAs varies from 10 minutes to several hours depends majorly on the property of domain II^10^. Some Aux/IAA proteins carrying mutations in domain II have a longer half-life and are insensitive to auxin^2,5^. The C-terminal domains III and IV of Aux/IAA share homology with domains of ARF, which renders the polymerization of Aux/IAA and ARF thus inhibiting the ARF function^11,12^. These Aux/IAAs lacking at least one conserved domain are regarded as non-canonical Aux/IAAs^10^. The emergence of non-canonical Aux/IAAs seems to be an ancient evolution event and important for plant adaption to different environment, as evident that non-canonical *Aux/IAAs* are shown to be presented in the *Aux/IAA* gene family in various plants^13–18^. The mechanism of non-canonical Aux/IAA proteins function is gaining attention. Two independent groups showed that non-canonical Aux/IAAs, IAA32, IAA33 and IAA34, act on the high concentration of auxin, and in a separate pathway than the canonical SCF^TIR^ pathway^19,20^. In rice, OsIAA26 with amino acids substitution in the degron in domain II works downstream of the canonical Aux/IAA OsIAA9 to integrate auxin and ethylene signaling^21^. It is also plausible that the non-canonical Aux/IAAs work together with the canonical Aux/IAAs to integrate the auxin pathway with multiple signaling cascades (Fig. 1C)^20,21^. Therefore, the domain composition may reveal the functions and downstream mechanisms of the Aux/IAAs.

Previous studies indicated that auxin is critical nearly in every aspect of plant development processes, including cell division, embryogenesis, lateral root initiation, vascular bundle extension, leaf extension, flowering patterning, fruit ripening, apical dominance, tropic growth and stress resistance^15,22–25^. The functional mechanism of Aux/IAA in various biological processes in plant growth and deployment has been well summarized carefully as in a canonical auxin pathway, especially in the *Arabidopsis*^22,26,27^. Apart from the role of trapping ARF, Aux/IAAs may also act as a hub to integrate other environmental cues^28,29^. For example, the screening for the suppressor of the phytochrome chromophore-deficient mutant *hy2* isolated a dominant *shy2/iaa3* mutant, suggesting the *Aux/IAAs* participating in the light signaling^30^. The mutation of *AXR2/IAA7* caused increased susceptibility to the necrotrophic fungi *Plectosphaaerella cucumerin*a and *Botrytis cinerea*^31^. Rice OsIAA10 was found to be hijacked by a *Rice dwarf virus* protein to enhance viral infection and pathogenesis^32^. Moreover, researches of *Aux/IAAs* in crops provided us new insights on the roles of *Aux/IAAs* in some species unique developmental processes which were largely neglected before, such as rice aerenchyma formation^33^ and maize tassels and ears formation^34^. In order to crack the complexity of elucidating the function of IAAs, their expression patterns are needed as a road map.

According to FAOSTAT (The Food and Agriculture Organization Corporate Statistical Database), barley is the fourth largest cereal crop (barley is grown in about 70 million hectares in the world) and is used for animal feed and malt. Genome-wide study of the barley *Aux/IAA* gene family has not yet been reported. We think that a comprehensive analyze of barley *Aux/IAAs* may help us analysis important agricultural traits associated with auxin in reverse genetics. As noticed by Youssef and Hansson^35^, auxin may play a major role in hormone crosstalk at the basal section of the spike. It would be interesting to test what are the roles of *HvIAA* in spikelet development.

The release of the barley genomic data^36^ enables us to isolate the *Aux/IAA* gene family in barley. We further performed a detailed analysis of sequence alignment, phylogenetic relationship, chromosome locations, gene structure, conserved domains, *cis*-acting elements, different expression patterns during 15 tissues based on the RNA-seq data, in response to NAA treatment condition and spike development. Our research revealed the expression of *Aux/IAAs* during barley spike development and the expressional response to exogenous auxins. These results provide clues for the functional characterization of *HvIAA* involved in the development of barley spike.

## Materials and methods

### Identification of *Aux/IAA* gene family in barley

*Arabidopsis* AtIAAs and rice OsIAAs protein sequences were downloaded from The *Arabidopsis* Information Resource (TAIR) (https://www.arabidopsis.org/)^13^ and Rice Genome Annotation Project (RGAP) (http://rice.plantbiology.msu.edu/)^37^, respectively. The barley protein and nucleotide sequences were obtained from the plant genomics database Phytozome (https://phytozome.jgi.doe.gov/pz/portal.html)^36^. The HMM (Hidden Markov Model) file was constructed based on the multiple sequence alignment of rice Aux/IAA protein by HMMER 3.0 software, and *Aux/IAA*s were queried in barley protein database (E-value less than e-10)^38,39^. A total of 42 barley proteins identified in this initial search were analysed using the HMM profiles of the Aux/IAA protein family Pfam02309 (AUX/IAA Superfamily: cl03528) with E-value less than e-5. As some ARF protein family proteins share conserved C-terminals with Aux/IAA family proteins (discussed in the introduction), the initial found 42 barley proteins consist some ARF proteins with conserved B3 DNA binding domain (pfam02362) and AUX/RESP domain (pfam06507)^40–42^. We eliminated these proteins as well as the redundant proteins and retained 36 proteins for further study.

### Primary sequence analysis

The isoelectric points, protein molecular weights and amino acids were obtained from ProtParam (https://web.expasy.org/protparam/)^43^, the Intro position was downloaded from Phytozome (http://www.phytozome.net)^44^. The Open Reading Frame (ORF) were obtained from the Sequence Manipulation Suite ORF Finder (http://www.bioinformatics.org/sms2/orf_find.html)^45^.

### Phylogenetic analysis

The phylogenetic tree was constructed with the Aux/IAA proteins from barley, rice, and *Arabidopsis* by MEGA 7.0 using the Neighbor Joining (NJ) method with 1,000 bootstrap replicates^46^, and modified with iTOL (https://itol.embl.de/itol.cgi).

### Analysis of gene structure and conserved motifs

For exon/intron structure of *HvIAAs* analysis, the CDS and protein sequences corresponding to each predicted gene were downloaded from Barley Genome Database Annotation on the Phytozome website. Multiple sequences alignments with barley (*Hordeum vulgare* L.) were conducted by Clustal W and Clustal X version 2.0 program of Jalview 2.11.0 software with Defaults^47,48^. The Multiple Expectation Maximization for motif Elicitation (MEME, http://meme-suite.org/tools/meme) tool was used to predict conserved motifs of HvIAA proteins^49^. The gene structure and conserved motifs were generated with TBtools software^50^.

### Analysis of chromosome locations and *cis*-acting elements

Chromosome physical position of *HvIAAs* was obtained from Phytozome^36^, using MapGene2Chrom web v2 (http://mg2c.iask.in/mg2c_v2.0/) to draw chromosome physical map. The identified CDS sequence of the *HvIAAs* was downloaded from Phytozome, and translated into protein sequences, using Muscle program alignment, and then introduced into the DnaSP 6 software to calculate the Ka and Ks values among the sequences of paralogous genes^51^. Super Circos was generated using TBtools software. The *cis*-elements in the 2,000 bp sequences upstream of the coding sequences were analyzed by Plant CARE databases (http://bioinformatics.psb.ugent.be/webtools/plantcare/html/).

### Expression analysis of barley *Aux/IAA* genes

Raw datasets were obtained from the BARLEX (Barley Genome Explorer)^36^ (https://apex.ipk-gatersleben.de/apex/f?p = 284:10:1811619314937)^52^. These data were applied to analyze *HvIAAs* expression profiles in different tissues. Heatmap was generated with the gplots package in R (https://www.r-project.org/). It showed the expression differences based on the FPKM values, which were normalized by log_2_^(FPKM+1)^ transform. Hierarchical clustering algorithms was used for recognition of similar patterns in expression files^53^.

### Plant growth, tissue collection and treatment

The spring barley cultivar of Gold Promise was grown in the greenhouse of Shanghai Jiao Tong University under 16 °C/14 °C day/night, 16 h/8 h light/dark, and 50% relative humidity. For NAA (1-Naphthaleneacetic acid) treatment, one-week-old seedling was sprayed with 5 nM NAA, and then the seedling was sampled at 0.5 h, 1 h, 2 h and 4 h after spraying. Seedlings were sprayed with DMSO (dimethyl sulfoxide) as a control (CK). To detect *HvIAAs* expression during the barley spike development, five stages were collected: two-week-old seedling (SD), the double ridge stage (DR), the lemma primordium stage (LP), the stamen primordium stage (SP), the awn primordium stage (AP) and the white anther stage (WA)^54,55^. The developmental stages were determined by dissection under a stereomicroscope. All samples were frozen in liquid nitrogen and stored at −80 °C until RNA extraction.

### Quantitative RT-PCR analysis

Barley tissues total RNA were extracted with TRIZOL reagent (Invitrogen), then reverse transcription reaction was carried out using a PrimeScript RT reagent kit with gDNA eraser (Takara), according to the manufacturer’s instructions. SYBR Green SuperReal PreMix Plus (TIANGEN) kit was applied for quantitative RT-PCR(qRT-PCR) experiments using CFX96 Real-time PCR machine (Bio-Rad). *HvACTIN* (HORVU5Hr1G039850.3) was used as an internal control^56^. Three biological repeats with three technical repeats were performed (primers used are listed in Supplementary Table S5).

## Results

### Identification of the *Aux/IAA* gene family in barley genome

Supplementary Tablestified from the barley genome using Hidden Markov Model (HMM) methods^38,39^. HvIAAs were designated as HvIAA1-HvIAA36 according to their physical position on barley each chromosome. We provided the gene characteristics including physical position, ORF (Open Reading Frame) sequence size, amino acid length, molecular weight (Da), isoelectric points (PI) and intron numbers. The number of amino acid length of the predict HvIAA proteins ranged from 96 (HvIAA21) to 772 (HvIAA34). The molecular weight of the HvIAA proteins differed from 10819.43 (HvIAA21) to 86193.2 (HvIAA34) Da, and the PI of the HvIAA proteins varied from 4.5 (HvIAA21) to 9.56 (HvIAA23) (Supplementary Table S1).

### Phylogenetic tree of the HvIAA proteins in barley

**Figure 2:**
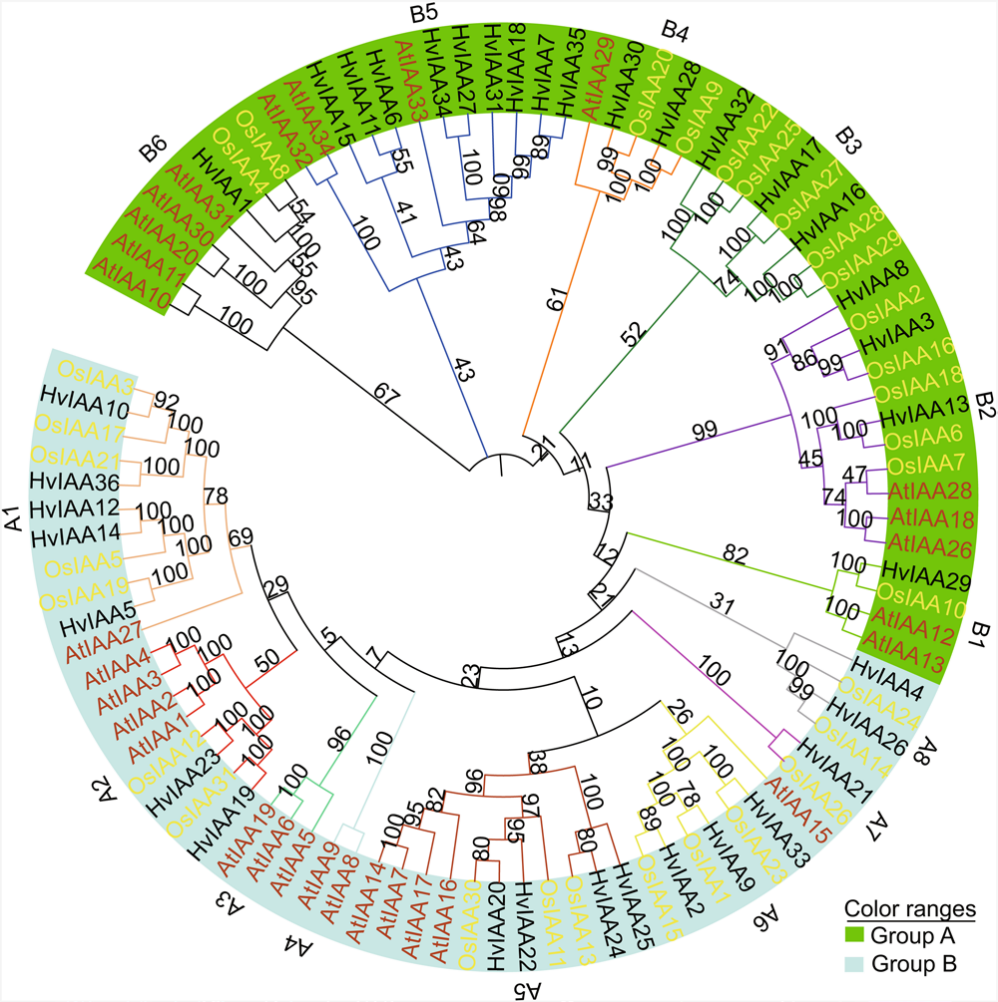
Phylogenetic tree of Aux/IAA proteins from barley, *Arabidopsis* and rice. The full-length amino acid sequences of 36 barley and 29 *Arabidopsis* Aux/IAA proteins combine with 31 Aux/IAA proteins from rice are aligned by ClustalW, and the neighbor-joining tree is constructed using MEGA7.0 with 1000 bootstrap replicates. Two groups A and B are the highlight in blue and green colors. Barley, rice and *Arabidopsis* are replaced by black, yellow and brown representing the Aux/IAAs, respectively.

To examine the phylogenetic relationships among the Aux/IAAs from barley, rice and *Arabidopsis* (based on 31 Aux/IAA protein sequences in rice and 29 Aux/IAA protein sequences in *Arabidopsis*), phylogenetic evolutionary trees of 96 Aux/IAA protein sequences are constructed using MEGA7.0 software^46^. According to the classification of rice and *Arabidopsis*^13,57^, 96 Aux/IAAs are divided into A and B groups with the well-supported branch. Based on the evolutionary, the classification of Aux/IAAs in *Arabidopsis* and rice is consistent with previous reports. There are 17 HvIAAs distributed in group A, while another 19 are found in group B (Fig. 2). Group A and B could be further subdivided into 8 and 6 subgroups (A1-A8 and B1-B6), respectively, with varying degrees of bootstrap support.

Subgroup A3 and A4 may specific to dicots, as they do not contain rice and barley IAAs. Subgroup A3 contains AtIAA5, AtIAA6, and AtIAA19. The Arabidopsis *iaa5/6/19* triple mutants line has a minor defect in stomatal movement under drought stress^58,59^. The missing of A3 subgroup in monocot indicate that the dumbbell stomata apparatus in monocot does not require the A3 subgroup IAAs paralog. Subgroup A4 contains AtIAA8 and AtIAA9, which were reported to regulate vasculature formation, adventitious root formation, and lateral root elongation^60–62^. As IAAs are essential for regulating lateral roots and adventitious roots both in monocot and dicots^63^, the absence of subgroups A4 IAA function may be compensated by other IAAs in other subgroups. For instance, OsIAA11 and OsIAA13 from subgroup A5 were reported in regulating lateral roots in rice^63,64^. We also identified subgroup A7, A8 and B3 IAAs as monocot specific. In our phylogeny tree, the subgroup A7 contains only two members, HvIAA21 and OsIAA26, which are also the smallest proteins in the Aux/IAA family. OsIAA26 was shown to be important for rice root elongation and the protein abundance is indirectly regulated by TIR^SCF1^-IAA signaling^21^. There is no data directly deciphering the function of A8 groups IAAs. However, studies suggested that the rice A8 groups *IAA* genes OsIAA14 and OsIAA24 are regulated by root development regulators, Crown Rootless1 (CRL1), MADS- box transcription factor (OsMADS25), and Nonexpressor of Pathogenesis-Related Genes1 (OsNPR1), indicating that this group of IAAs may function specifically in root development^65–67^. Likewise, there is no direct data to infer the function of B3 IAAs. Interestingly, the subgroup B5 only contains barley and Arabidopsis IAAs. It is intriguing to ask whether this group of IAAs is related to the xeromorph of barley and Arabidopsis. All the three Arabidopsis members, IAA32, IAA33and IAA34 were shown to act on high auxin level^19,20^.

### Chromosomal locations of *HvIAA* genes

The physical location of 35 *HvIAAs* determines the location on the chromosome. 35 *HvIAA*s (97.2%, 35/36) locate unevenly on 7 chromosomes, *HvIAA36* was not able to be mapped to chromosome (Supplementary Table S1). ChrUn is composed of sequence fragments originating from BAC (bacterial artificial chromosome) overlap clusters not placed in the Hi-C (high-throughput/resolution chromosome conformation capture) map-, or gene-bearing fragments of BAC sequences and Morex WGS (whole genome shotgun) contigs selected in addition to the non-redundant sequence^68^. The nine *HvIAAs* are located on chromosome 5, two on chromosome 2 and 4, five are located on chromosome 1, eight on chromosome 3, three on chromosome 6, and six on chromosome 7, respectively (Supplementary Fig. S1).

**Figure 3:**
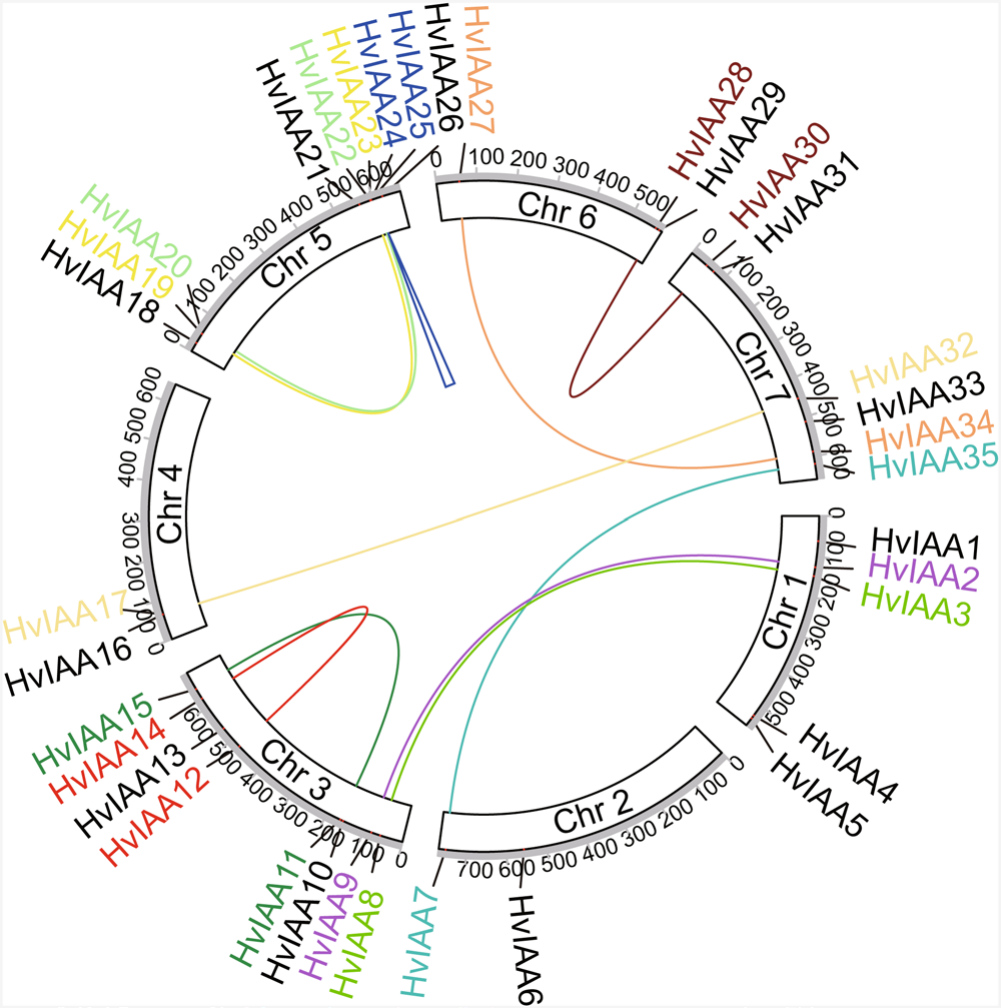
Chromosomal locations and duplication events of *HvIAA* members in barley genome Colorful lines and blue triangle represent gene duplications.

Phylogenetic analyses can often be used to uncover the duplication events that led to the generation of large sets of tandemly duplicated genes^69^. In this study, 36 HvIAAs only formed 8 sister pairs (Supplementary Fig. S2) with strong bootstrap support (>97%), at least 11 paralogs of HvIAAs might have undergone gene duplication, which may be caused by segment replication and tandem replication events (Fig. 3; Supplementary Fig. S2)^70^. Segment duplication leads to many homologies of *HvIAAs* between the chromosomes.

In order to explore which type of selective pressure determines the divergence process of *HvIAA* after replication. Ka/Ks is the ratio of nonsynonymous substitution (Ka) to synonymous substitution (Ks), the Ka/Ks substitution ratio is used to assess the coding sequences of 11 pairs of *HvIAAs*. A Ka/Ks ration greater than 1 represents positive selection, a ratio of 1 represents neutral evolution and a ratio less than1 represents purifying selection^71^. We found that nine out of the eleven *Aux/IAA* paralog pairs had the ratio of Ka/Ks among 11 pairs genes was less than 1(Supplementary Table S2), suggesting a purified selection that favors synonymous substitutions than nonsynonymous substitutions to prevents the change of an amino acid residues had occurred following the duplications.

### Gene structure and conserved motif analysis of the barley *Aux/IAA* genes

**Figure 4:**
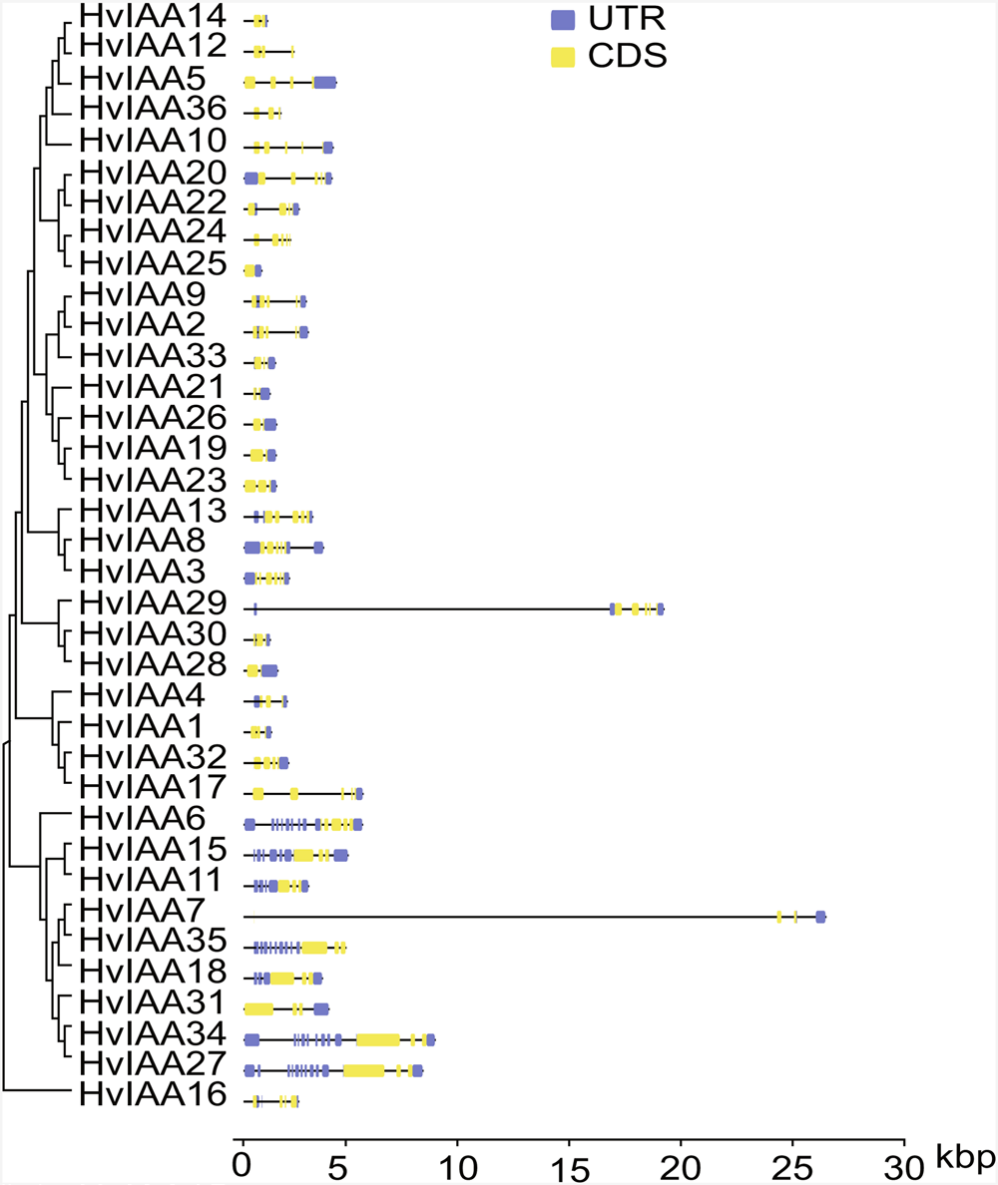
Gene structure analysis of barley *HvIAAs* according to a phylogenetic relationship. The UTR, exons, introns, are represented by blue boxes, yellow boxes and black lines, respectively.

In the previous research, gene structure diversity provides the main impetus for the evolution of polygenic families^72–74^. Therefore, the gene structural diversity of *HvIAAs* is further studied with exon/intron analysis. The result shows that the sequence length and the number of introns (exons) of 36 *HvIAAs* vary greatly. The number of introns ranges from 0 to 13, of which *HvIAA25* has a complete lack of introns, and *HvIAA35* has most introns, with 13 introns. Additionally, some *HvIAAs* in the same phylogenetic subgroup had the same number of exons such as *HvIAA2* and *HvIAA9*, *HvIAA34* and *HvIAA27*. Interestingly, these two pairs of *HvIAAs* have similar gene sequence length (Fig. 4).

**Figure 5:**
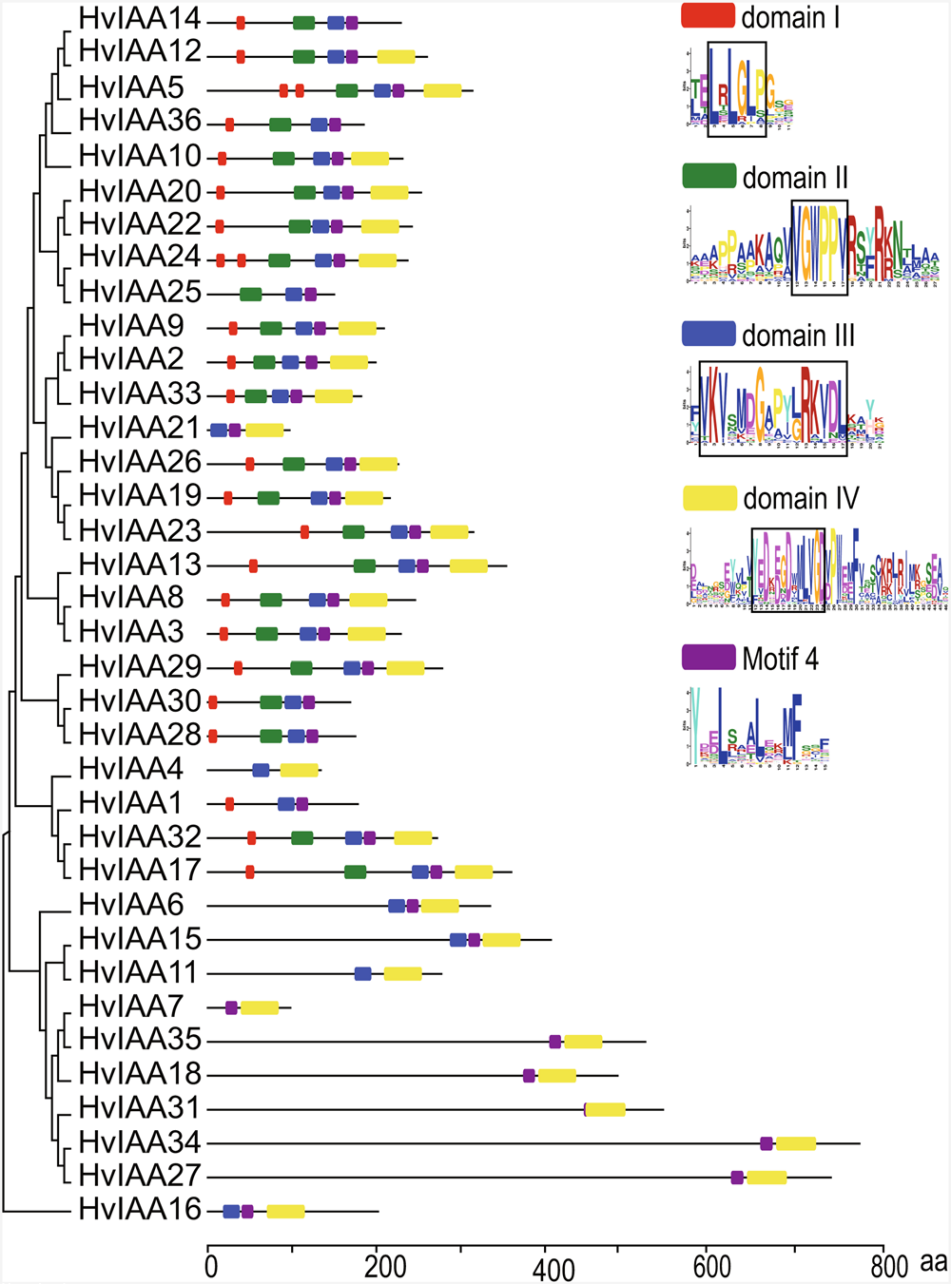
The conserved motifs of HvIAAs according to a phylogenetic relationship. All motifs are identified by MEME with the complete amino acid sequences of HvIAAs. Different motifs are represented by different box colors. The bits indicate amino acid conservation in each position. The conserved sequences are highlighted with black boxes on each domain. Red, green, blue, yellow and purple boxes represent motif 5, motif 3, motif 2, motif 1 and motif 4, respectively.

The motif discovery (classic mode) of the MEME online tool^49^ is used to analysis motif distribution of barley Aux/IAA protein. 5 motifs are found in most of the HvIAA proteins. According to our MEME analysis, 4 conserved motifs of HvIAA are corresponding to the four conserved domains of Aux/IAA protein. Motif 1, 2, 3 and 5 are corresponding to domain IV, III, II, I, respectively. 18 HvIAA proteins have all the four conserved domains, while the rest 18 proteins lack at least one conserved domain, belonging to non-canonical Aux/IAAs. Some of the HvIAA protein sequences lack conserved motifs, such as 6 HvIAA proteins (16.7%, 6/36) do not have motif 1, 6 HvIAA proteins (16.7%, 6/36) do not have motif 2, 13 HvIAA proteins (36.1%, 13/36) do not have motif 3, and 23 HvIAA proteins (63.7%, 23/36) have motif 5. Peculiarly, HvIAA4, HvIAA7, HvIAA18, HvIAA27, HvIAA31, HvIAA34, HvIAA35 proteins contain only one motif (Fig. 5). Multiple alignment analysis of 36 HvIAA protein sequences in barley, two nuclear localization signals (NLSs), multiple phosphorylation sites and βαα motif are found in most of identified HvIAA proteins (Supplementary Fig. S3).

### Analysis of *cis*-elements in *HvIAA* genes

In order to identify the potential regulatory elements response to auxin. We used the Plant Care database to identify the *cis*-acting elements existing in the promoter region of *HvIAA*s. The detailed information of *cis*-acting elements at the promoter regions was listed in (Supplementary Table S3), except for one gene (*HvIAA6*, no clear promoter sequence). According to the previous study, we classify them into six major functional categories; development/tissue specificity, promoter/enhancer element, light response, circadian control, stress and hormone response^75^. The result reveals that two types of *cis*-acting elements are involved in hormone response and related to auxin response, including AuxRRs and TGA-element (Marked in red color in Supplementary Table S3). It can be inferred that *HvIAAs* can response to exogenous auxin^76^. Moreover, CAAT-box and TATA-box are ubiquitously presented in the promoter/enhancer element of *HvIAAs* (Supplementary Fig. S4), it is one of the binding sites for RNA polymerase and it also determines the initiation of gene transcription and its efficiency. Therefore, CAAT-box and TATA-box may play an important role in the initiation of controlling *HvIAAs* expression time and degree.

### Expression analysis of *HvIAA* genes in different tissues

**Figure 6:**
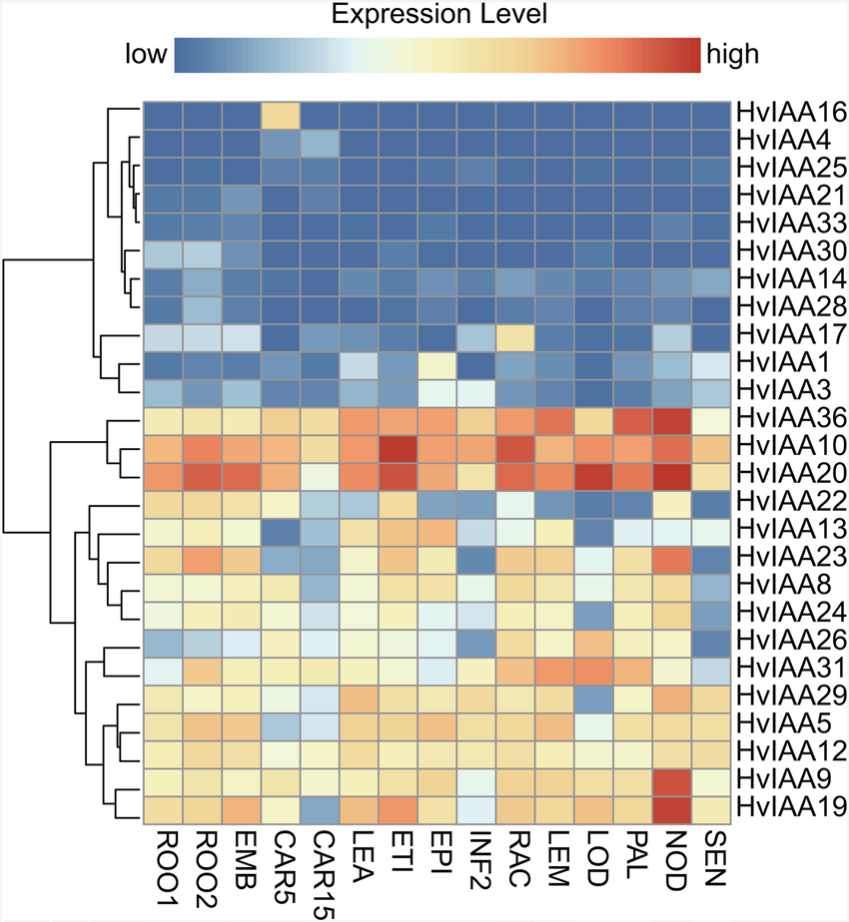
Hierarchical clustering of *HvIAAs* expression in various tissue. FPKM values were normalized by log_2_^(FPKM+1)^ transform to represent color scores. ROO1, roots from seedlings (10 cm shoot stage); ROO2, roots (28 DAP); EMB, 4 day embryos; CAR5, developing grain (5 DAP); CAR15, developing grain (15 DAP); LEA, shoots from seedlings (10 cm shoot stage); ETI, etiolated seedling, dark cond. (10 DAP); EPI, epidermal strips (28 DAP); INF2, developing inflorescences (1–1.5 cm); RAC, inflorescences, rachis (35 DAP); LEM, inflorescences, lemma (42 DAP); LOD, inflorescences, lodicule (42 DAP); PAL, dissected inflorescences, palea (42 DAP); NOD, developing tillers, 3rd internode (42 DAP); SEN, senescing leaves (56 DAP).

Analysis of gene family expression patterns provides information for studying their functions^77,78^. The results of expression analysis show that 26 *HvIAAs* have different expression patterns in 15 different developmental stages based on RNA-seq data, and the other 10 *HvIAAs* have no data (Fig. 6; Supplementary Table S4). Hierarchical clustering was used to cluster the genes with similar expression patterns, which divided the *HvIAA* into three clusters. The *HvIAAs* in Cluster I (including eleven members) display low expression levels in most tissues, whereas *HvIAA16* and *HvIAA17* have high expression in the specific tissues compared with other genes in Cluster I. In Cluster II (including three members) genes are general highly expressed in different tissues, for example, *HvIAA20* shows a strong expression level in developing tillers (NOD) compared with other 14 tissues. *HvIAA* transcripts are less abundant in developing grain (CAR 15) and senescing leaves (SEN). In Cluster III (including twelve members), genes are expressed at a moderate level.

*HvIAAs* in Cluster I is merely mildly expressed, suggesting that these genes might be less required in those tissues. Remarkably, *HvIAA16* is only expressed in developing grain (CAR 5). The expression of genes in Cluster II, in etiolated seedling (ETI), inflorescences, rachis (RAC) and developing tillers (NOD) was higher than other genes in this group. The expression of genes in 15 different tissues of Cluster III shows a fluctuating trend. For instance, the expression of *HvIAA19* is highest in developing tillers (NOD), but it is the lowest in developing grain (CAR 15). *HvIAA19* may play a key role in development tillers (NOD) in the barley development. These results suggest that these genes have specific functions in different tissue and development processes.

### Expression of *HvIAAs* genes in the spike development and response to NAA treatment

**Figure 7:**
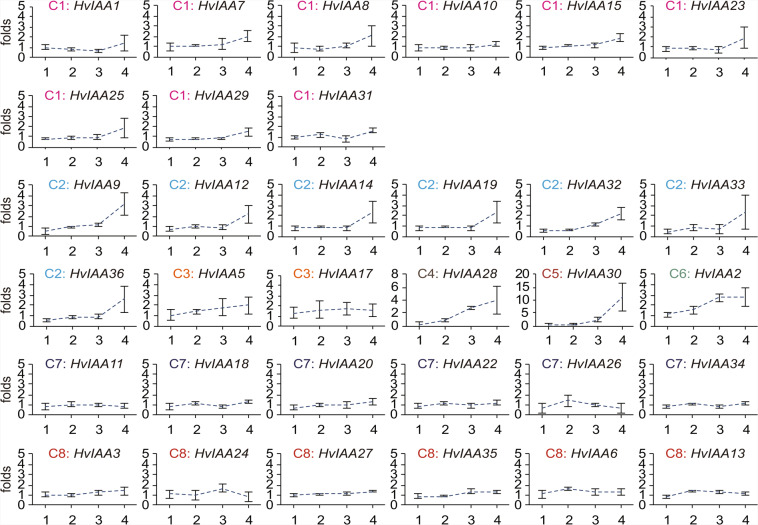
Expression profiles of the *HvIAAs* in response to NAA treatment. qRT-PCR analyses are used to assess *HvIAAs* transcript levels in the one-week seedlings sampled at 0.5, 1, 2 and 4 h after spraying 5 nM NAA. The number on x-axis indicate hours after NAA treatment. These genes are divided into C 1– C 8. The abscissas 1, 2, 3, and 4 represent 0.5 h, 1 h, 2 h and 4 h, respectively.

To investigate whether *HvIAAs* respond to auxin, the expression levels of *HvIAAs* are evaluated in the barley one-week seedling by qRT-PCR under NAA treatment. According to expression pattern after NAA treatment, then clustered using hierarchical clustering algorithms, these genes are divided into C1- C8. The data showed that the transcript levels of group C2, 4, 5, and 6 of the *HvIAAs* are upregulated at 4 h after NAA treatment. Especially, *HvIAA30* is upregulated over ten-fold at 4 h, *HvIAA2* and *HvIAA*30 are upregulated over two-fold at 2 and 4 h (*HvIAA4*, *HvIAA16* and *HvIAA21* are not detectable). Interestingly, *HvIAA33* is decreased at 0.5 h after NAA treatment (Fig. 7).

**Figure 8:**
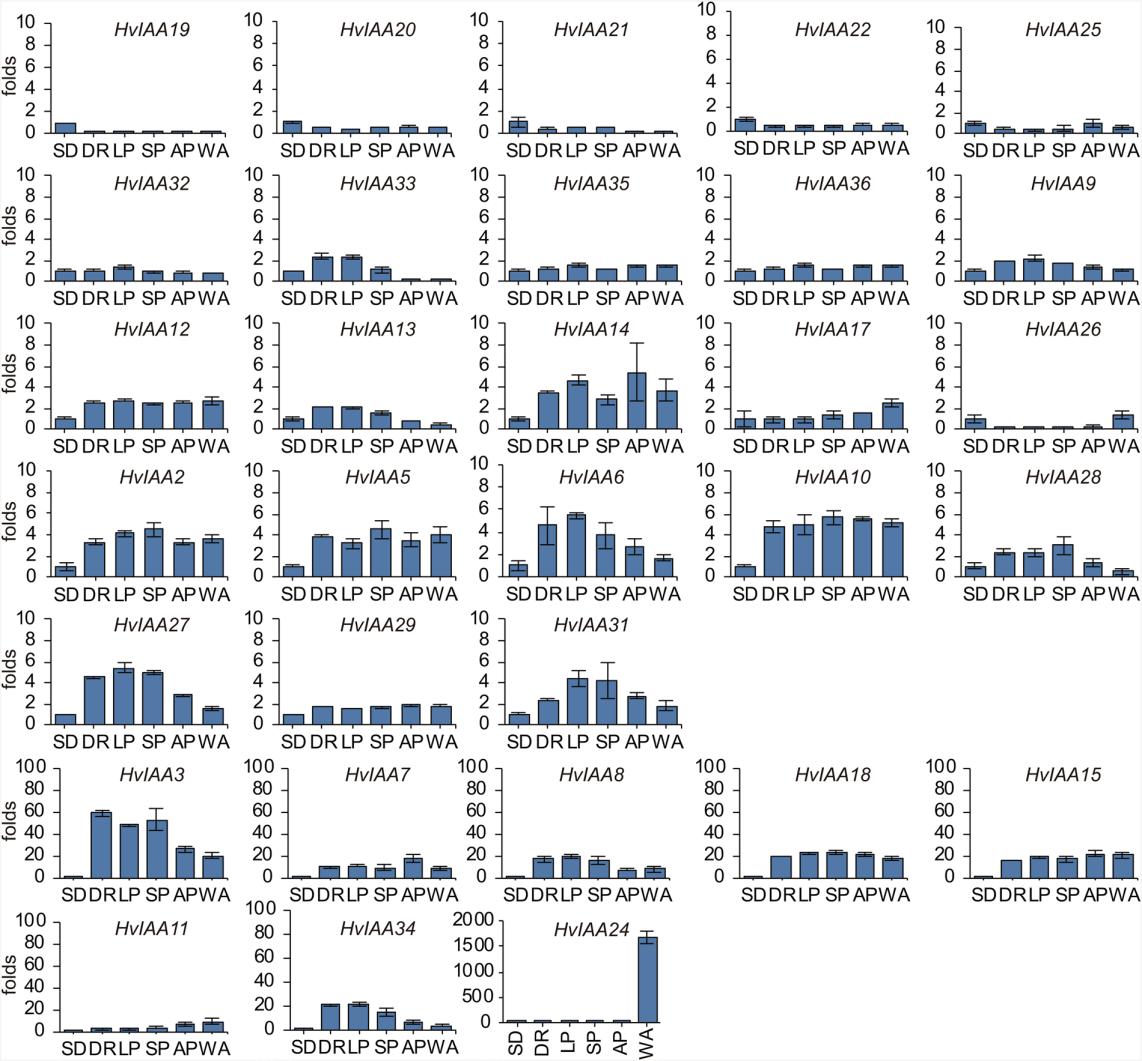
Expression profiles of the *HvIAAs* during barley spike five developmental stages. qRT-PCR analyses are performed using RNA generate from barley spike developmental stages. SD: two-week-old seedling; DR: double ridge stage; LP: lemma primordium stage; SP: stamen primordium stage; AP: awn primordium stage; WA: white anther stage.

To investigate the expression of *HvIAAs* associated with barley spike development, the transcript levels of each *HvIAA* is monitored in the spike development and their expression patterns are analyzed using qRT-PCR. The result shows that most of the *HvIAAs* are detected in the barley spike development compare with two-week-old seedling (SD) (*HvIAA1*, *HvIAA4*, *HvIAA16* and *HvIAA30* are not detect data), such as double ridge stage (DR), lemma primordium stage (LP), stamen primordium stage (SP), awn primordium stage (AP) and white anther stage (WA) (Supplementary Fig. S5). However, some *HvIAAs* genes display a high expression during barley spike development. For example, *HvIAA3*, *HvIAA7*, *HvIAA8* and *HvIAA18* are highly expressed in the DR, LP, SP, AP and WA. Interestingly, *HvIAA24* shows the highest expression level in WA. *HvIAA34* displays a higher expression level in the DR, LP and SP than in the other stages (Fig. 8).

## Discussion

Here, we identified 36 *Aux/IAAs* from the barley genome, each of them has at least one conserved Aux/IAA domain. Intriguingly, barley (5.1 GB) has a large difference in genome size compare to rice (430 MB) and *Arabidopsis* (135 MB), but the numbers of *Aux/IAA* members are very close^13,58^. In contrast to the higher plants, *Marchantia* and *Physcomitrella* have only one and three *Aux/IAAs,* respectively^79–81^.

This implies that *Aux/IAA* gene families are expanded when plants start to conquer the land during evolution and the current number for higher plants is essential for their well-being. Nevertheless, only a few of the *Aux/IAAs* loss-of-function mutants in *Arabidopsis* have only mild phenotypes^82^. Investigation of additional species outside of *Arabidopsis* is recommended for a better understand of the true biological functions of *Aux/IAAs*^82^.Consistent with the finding in *Arabidopsis*, there is no premature stop-codon found in barley *Aux/IAA* genes, suggested that they are not likely pseudogenes^57^.

The 36 barley, 29 *Arabidopsis*, and 31 rice Aux/IAA proteins sequences were used to construct evolutionary tree by using Neighbor-Joining method.The 36 HvIAAs can be classified into two groups, Group A (17 members) and B (19 members), which is consistent with the classification in rice^13^ and *Arabidopsis*^57^. We found that most of the *HvIAAs* are located at the distal ends of the chromosome. Similar findings on other barley gene families were also reported^53,83,84^. This may due to the fact that there are overall more abundant genes at both ends than in the middle of the chromosome in barley^36^. It is the meiotic homologous chromosome recombination mainly confined to the distal regions of all chromosomes that renders the uneven density of genes on cereal chromosomes and most of the genes concentrated in the distal regions^85^. Gene duplication events accelerate the rapid expansion and evolution of gene families. In this study, at least 11 pairs of *HvIAAs* undergone gene duplication (Fig. 3; Supplementary Fig. S2), including segment duplication and tandem duplication events. For instance *HvIAA2* and *HvIAA9* may be the products of genomic segment replication. *HvIAA24* and *HvIAA25* are the products of genomic tandem replication. Generally, this kind of genes arrangement is difficult to segregate via hybridization in breeding or research. These members belonging to the same clades usually share similar conserved motif and exon/intron, and they may have similar functions. Nine out of eleven pairs of the paralogous genes are Ka/Ks less than 1, indicating they have undergone purifying selection.

The domain I containing the LxLxLx motif (L, leucine, x represents any amino acid residue) is important for the IAAs as active transcriptional repressors. It couples the TOPLESS protein scaffold (TPL) and thereafter recruiting histone deacetylase to the ARF transcription factor. The Lego bricks structure transforms the ARF to a transcriptional repressor^7,86,87^. Nevertheless, there are abundant numbers of IAA lacking domain I in various species and they are also able to suppress the expression of auxin response genes. IAAs lacking domain I oligomerize with ARF to prevent the activation activity of ARF^87^. In this case, Aux/IAA is an indirect transcription activator acting through modulating the free ARFs level. Besides, IAAs were shown to repress ARF activity without dimerizing with ARF^88^. In our study, we identified HvIAA25 from A5 subgroup, HvIAA21 from A7 subgroup, HvIAA4 from A8 subgroup, HvIAA13 from B2 subgroup, HvIAA16 from B3 subgroup and HvIAA6, HvIAA7, HvIAA11, HvIAA15, HvIAA18, HvIAA27, HvIAA34 and HvIAA35 from B5 subgroup are lacking domain I (Fig. 2 and Fig. 5). However, how different in the transcription repression activity of the barely IAAs with/without domain I need to be further checked.

The core sequence of Domain II with GWPPV/I is the target site for ubiquitination degradation of Aux/IAA protein by interaction with TIR1 of SCF^TIR1^ complex. The absence of domain II in AtIAA20 and AtIAA30 leads to the longer half-life of the proteins comparing to the canonical Aux/IAA proteins, and overexpression of either of them disturbs auxin physiology and causes auxin-related aberrant phenotypes^89^. Previous studies showed non canonical IAA proteins lacking domain II are not subject to the degradation by the SCF^TIR1^ protein complex and stabilized by the phosphorylation of IAAs on high auxin level^19,20^. In our study, we identified HvIAA21 from A7 subgroup, HvIAA4 from A8 subgroup, HvIAA6, HvIAA7, HvIAA11, HvIAA15, HvIAA18, HvIAA27, HvIAA31, HvIAA34 and HvIAA35 from B5 subgroup, and HvIAA1 from B6 subgroup and HvIAA16 from B3 subgroup are lacking domain II (Fig. 2 and Fig. 5). They may share the common function mechanism as these reported non canonical IAAs. Similar with lacking domain II, mutations in the conserved motif GWPPV/I motif in the domain II, such as AtIAA31 in Arabidopsis and OsIAA4 and OsIAA10 in rice, and the Arabidopsis mutants *shy2–2* and *shy2–3* (IAA3), *iaa18-1* (IAA18)*, arx2-1* (IAA7), *arx3-1* and *arx3-3* (IAA17), also stabilize the protein and the overexpression of these genes exhibits the typical auxin-related aberrant phenotypes such as dwarfism, increased tiller angles, reduced gravity response^89–92^. In our study, similar dominant mutation in IAA domain II if it has domain II was not found in the barley genome (Supplementary Fig. S3).

Domain III and IV are conserved between the Aux/IAA and ARF protein families, they are mainly responsible for the homo- and hetero-dimerization among the Aux/IAA proteins and ARFs to inhibit the transcription of auxin-responsive genes^11^. Additionally, the βαα motif in domain III composed of a β-sheet and two α-helices (α 1 and α 2) is critical for the dimerization of Aux/IAA protein^7^. Most of the HvIAA proteins have two putative nuclear localization signals (NLS), the first one is composed of a bipartite structure, conserved double KR between domain I and domain II and basic amino acids in domain II, while the second one includes SV40-type NLS located in domain III^93^, assuming that HvIAA proteins may play a role in the nucleus. Furthermore, multiple phosphorylation sites are discovered in the HvIAA proteins, these different phosphorylation sites may regulate nuclear transportation (Supplementary Fig. S3)^94^. Domain III and domain IV in the C-terminal region, has well-known Phox and Bem 1 (PB1) protein-protein interaction domain that mediates homo- and heterodimerization^95^. In our study, we identified HvIAA7, HvIAA18, HvIAA27, HvIAA31, HvIAA34 and HvIAA35 from subgroup B5, are lacking domain III. HvIAA14 and HvIAA36 from subgroup A1, HvIAA25 from subgroup A5, HvIAA28 and HvIAA30 from subgroup B4, and HvIAA1 from subgroup B6, which are lacking domain IV. Genetic and biochemical data on their function are needed to further elucidate these non-canonical IAAs.

Gene expression gives hints for the function of the gene. In order to characterize the expression regulation of *HvIAAs*, we check cis-elements in the promoter regions of the *HvIAAs*. We found that the promoter regions of *HvIAAs* contain *cis*-elements related to development/tissue specificity, promoter/enhancer element, light response, circadian control rhythm, stress and hormone response (Supplementary Table S3), indicating that expression of *HvIAAs* is tailored to adapt the multiple functions in various biological processes. The result shows that 18 *HvIAAs* contains one or two auxin response elements, which may be regulated by auxin. *HvIAA8*, *HvIAA15*, *HvIAA20*, *HvIAA27* have two auxin response elements, and the other 18 *HvIAAs* do not have any auxin response elements (Supplementary Table S3). The presence of these *cis*-acting elements suggests that the *HvIAAs* play important roles in the early response of auxin in barley. Previous work shows that auxin and light, brassinolide and abiotic stress signal are mutually regulated^96–98^. *OsIAA1* and *HvIAA9*, *OsIAA6* and *HvIAA13* are in the same clades, and *cis*-acting elements analysis suggesting that canonical *HvIAA9* and *HvIAA13* contain TGA-element in their promoters, it can respond to exogenous auxin. Undoubtedly, several of *cis*-acting elements in the promoter regions of *HvIAAs* coordinate the regulate expression of the *HvIAAs* to facilitate their functions in barley development. It is helpful to validate the expressional pattern of *HvIAAs* experimentally to reveal the gene function of *HvIAAs*.

The diversity of the expression profiles of *HvIAAs* in tissues and developmental stages indicates that *HvIAAs* regulate multiple developmental processes. The expression pattern of HvIAA provides hints to further investigation of their biological function. For example, the high expression of *HvIAA20* in inflorescence (LOD) implies that *HvIAA20* may regulate inflorescence development. *HvIAA16* displays expression in developing grain (CAR5), which suggests that *HvIAA16* may be related to grain development. Furthermore, *HvIAA9*, *HvIAA19*, *HvIAA20* and *HvIAA36* have a high expression level in developing tillers (NOD), indicating that these four genes may together involve in developing tillers development (Fig. 6). In previous studies, *OsIAA9* promotes lateral root formation in rice, and mediates geotropic of deletion is associated with starch granule synthesis in root tips^99^. According to expression analysis and phylogenetic relation of *OsIAA9* with *HvIAA28* and *HvIAA30*, *HvIAA28* and *HvIAA30* may influence the root growth. *OsIAA11* inhibits the development of lateral roots and influences inflorescence genes in rice^100^. The expression profile and phylogenetic tree indicate that the subcluster including *OsIAA11*, *HvIAA20* and *HvIAA22* may probably participate in the root and inflorescence growth. The mutation of *AtIAA12* in *Arabidopsis* specifically affects embryonic development^101^.

Although *Aux/IAA* transcription was initially thought to be primary auxin responsive, different expression patterns in response to auxin were found among the gene family in various species^13,102,103^. In C 2, 4, 5 and 6 group, the expression levels are significantly upregulated at 4 h after NAA treatment. Especially, *HvIAA30* is upregulated over ten-fold at 4 h, *HvIAA2* and *HvIAA*30 are upregulated over two-fold at 2 and 4 h (Fig. 7). Our promotor analysis identified two auxin signaling transduction-related *cis*-elements presenting in the promoter regions of the 18 *HvIAAs* (Supplementary Table S3). The diversity of the numbers and locations of these *cis*-elements may partly explain the different expression patterns of *HvIAAs* under NAA treatment. However, *HvIAA2*, *HvIAA5*, *HvIAA10*, *HvIAA14*, *HvIAA19* and *HvIAA28* are not found the auxin-responsive elements in the promoter regions, the relative mRNA levels of these 6 genes increased at 4 h after the NAA treatment (Fig. 7). Similar results have been reported in previous research^104^. The transcript levels of the *OsIAA9*, *OsIAA19*, *OsIAA20* and *OsIAA31* are prominent upregulated under auxin treatment^13^, *HvIAA28*, *HvIAA5*, *HvIAA30* and *HvIAA19* are homologs of these four *OsIAA* genes, respectively (Fig. 2), and detect the expression of upregulated after NAA treatment.

Traits of barley spikelet largely contribute to the yield. Therefore, the investigation of genes regulating spikelet development may serve for the barley breeding aimed for improving yield. There are accumulating evidence that *Aux/IAA* genes involve in regulating inflorescence structure and thus spikelet in various species^34,35,105^. In the previous study, overexpression of *IAA1* in *Arabidopsis* significantly reduced cell length and cell number in inflorescences and leaves, and affected cell shape^105^. *BIF1* (*BARREN INFLORESCENCE1*) and *BIF4* (*BARREN INFLORESCENCE4*) encoded the Aux/IAA protein regulate the early steps required for inflorescence formation^34^. In our study, the expression of *HvIAA3*, *HvIAA7*, *HvIAA8*, *HvIAA18*, *HvIAA15* and *HvIAA34* are increased during the barley spike developmental process, suggesting that these 6 genes might play a key role in the development of barley spike. Interestingly, *HvIAA24* displays the highest expression level in the WA (floral organ differentiation was completed), implying that this gene may act in awn development. *HvIAA34* exhibits higher expression in the DR, LP and SP than in the other tissues, indicating that this gene may function specifically in DR, LP and SP stages during barley spike development (Fig. 8). Again, there is not likely *IAA* pseudogenes in barley as all of them are found expressed at least in one condition.

As in other plants, the *Aux/IAAs* could play important roles in the growth and development of barley. However, the biological function of the barley *Aux/IAAs* remains to be elucidated in detail. Our data on the phylogeny, gene structure, conserved motifs, chromosome locations, *cis*-acting elements and expression profiles provide essential clues for exploring the biological functions of the 36 *Aux/IAAs* in barley.

## Conclusion

In this study, we identified 36 *Aux/IAAs* in barley, similar number as that in rice and *Arabidopsis*. Half of them are canonical Aux/IAAs containing the typical four conserved IAA domains and the rest are non-canonical Aux/IAAs. We identified 14 expressional auxins responding *HvIAAs* and numbers of *HvIAAs* that may regulate spike development. Our findings would facilitate the functional study of *Aux/IAA* genes and molecular breeding of barley.

## Supplementary information

Supplementary Information.

Supplementary Table S1.

Supplementary Table S2.

Supplementary Table S3.

Supplementary Table S4.

Supplementary Table S5.
