# Supplementary material for: Genome-wide characterization and expression analyses of the auxin/indole-3-acetic acid (Aux/IAA) gene family in barley (Hordeum vulgare L.)
Source: Sci Rep. 2020 Jun 24;10:10242. doi: 10.1038/s41598-020-66860-7 (PMC7314776; doi:10.1038/s41598-020-66860-7)
Supplement: Supplementary file 1 — Supplementary Information. [file 41598_2020_66860_MOESM1_ESM.docx]

Genome-wide characterization and expression analyses of the *auxin/indole-3-acetic acid* (*Aux/IAA*) gene family in barley (*Hordeum vulgare* L.)

Qi Shi^1,2,3^; Yueya Zhang^3^; Vinh-Trieu To^3^; Jin Shi^3^; Dabing Zhang^1,2,3,4*^; Wenguo Cai^3,5*^;

1. Key Laboratory of Ministry of Education for Conservation and Utilization of Special Biological Resources in Western China, Ningxia University, Ningxia 750021, China
2. College of Life Science, Ningxia University, Yinchuan, Ningxia 750021, China
3. Joint International Research Laboratory of Metabolic and Developmental Sciences, State Key Laboratory of Hybrid Rice, School of Life Sciences and Biotechnology, Shanghai Jiao Tong University, Shanghai 200240, China
4. School of Agriculture, Food and Wine, University of Adelaide, Urrbrae, SA 5064, Australia.
5. Flow Station of Post-doctoral Scientific Research, School of Life Sciences and Biotechnology, Shanghai Jiao Tong University, Shanghai 200240, China.

The authors declare no conflict of interest.

* To whom correspondence should be addressed: Dabing Zhang zhangdb@sjtu.edu.cn; Wenguo Cai wenguo.cai@sjtu.edu.cn

**Supplementary Table S1** *Aux/IAA* gene family in barley.

**Supplementary Table S2** Ka and Ks calculations of the *HvIAA* gene pairs

**Supplementary Table S3** *Cis*-acting elements found in the promoter region of *HvIAAs*

**Supplementary Table S4** The FPKM values of *HvIAA* genes in different tissues.

**Supplementary Table S5** Sequences of primers used in this study.


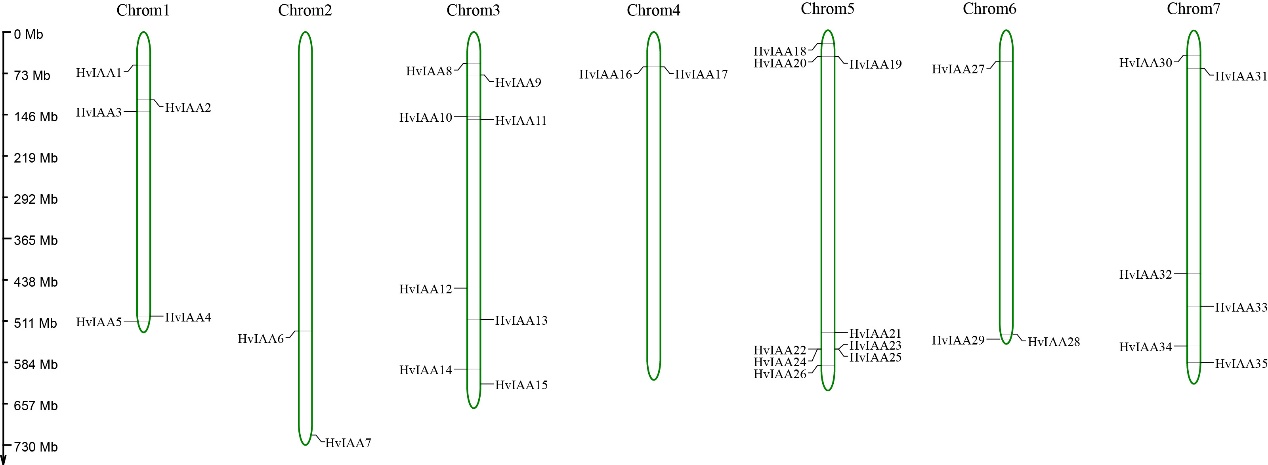


**Supplementary Fig. 1** The distribution of 35 *HvIAAs* on seven barley chromosomes. The name of each chromosome is displayed at the top of the corresponding chromosome. Each approximate location of *HvIAAs* is marked with chromosome and gene names showed on the left and right bars.


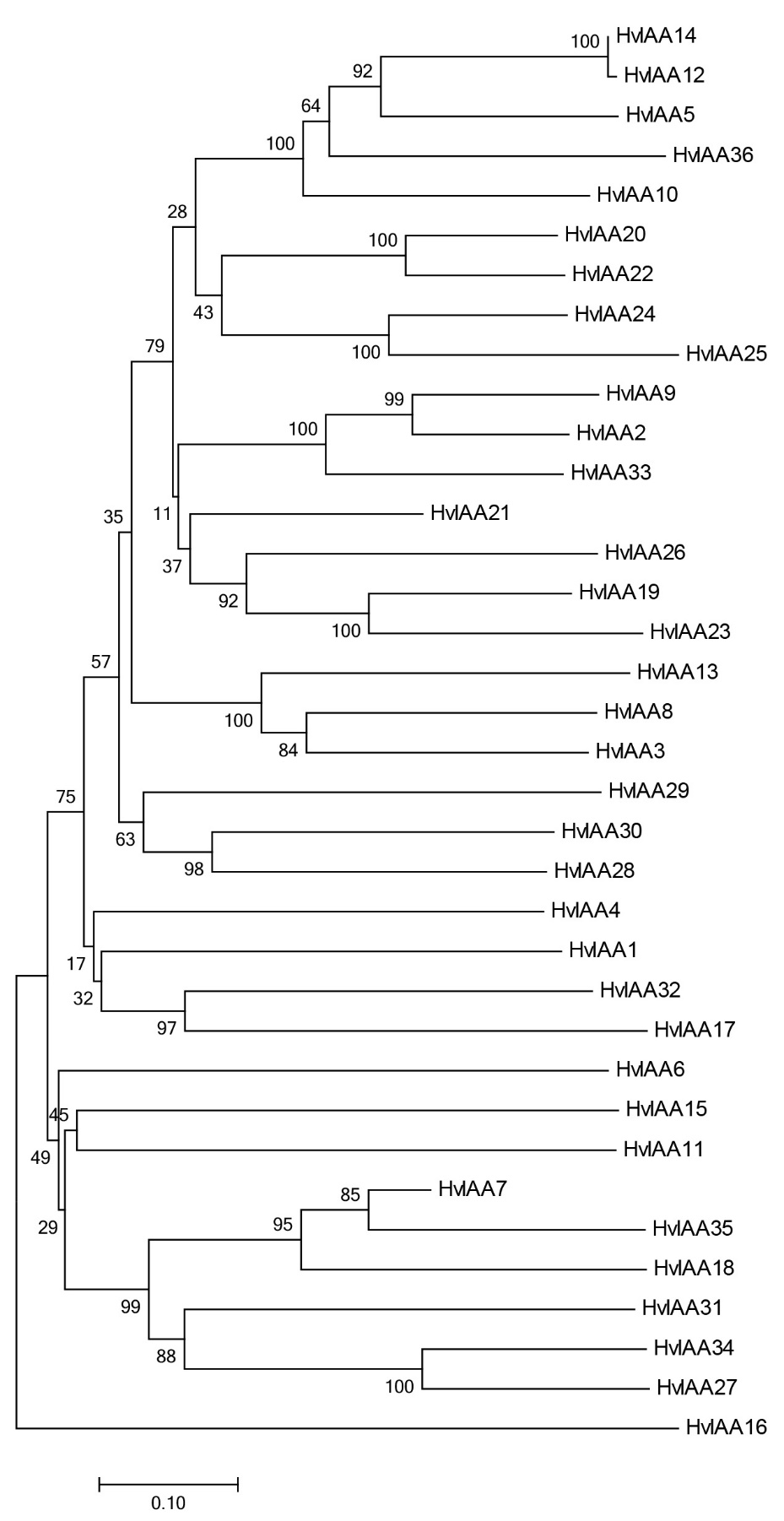


**Supplementary Fig. 2** The phylogenetic relationships among the HvIAA proteins.

**
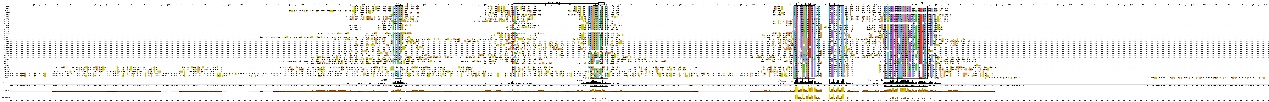
**

**Supplementary Fig. 3** Sequence alignment of HvIAA family proteins. The HvIAA protein sequences were aligned using Jalview software of Clustal W and Clustal X version 2.0 program and manual correction, obtained domains I to IV, Conserved domains are indicated by roman numerals (region marked by a *black underscore* in **Fig. S3**). Two NLSs were represented by *open boxes.* And one *βαα* motif was shown by *black bar*. Phosphorylation sites are emphasized by *black solid circle.*

**
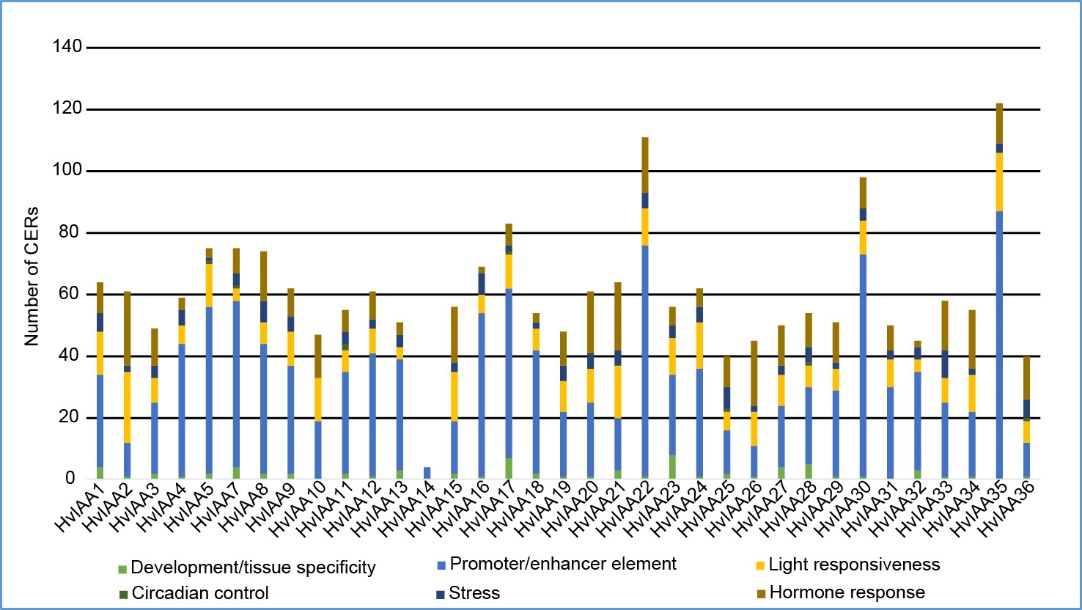
**

**Supplementary Fig. 4** *Cis*-acting regulatory elements in *HvIAAs. Cis*-acting regulatory elements (CREs) found in the promoter region of *HvIAAs*. The detail information of CREs was shown in the (**Table S3**).
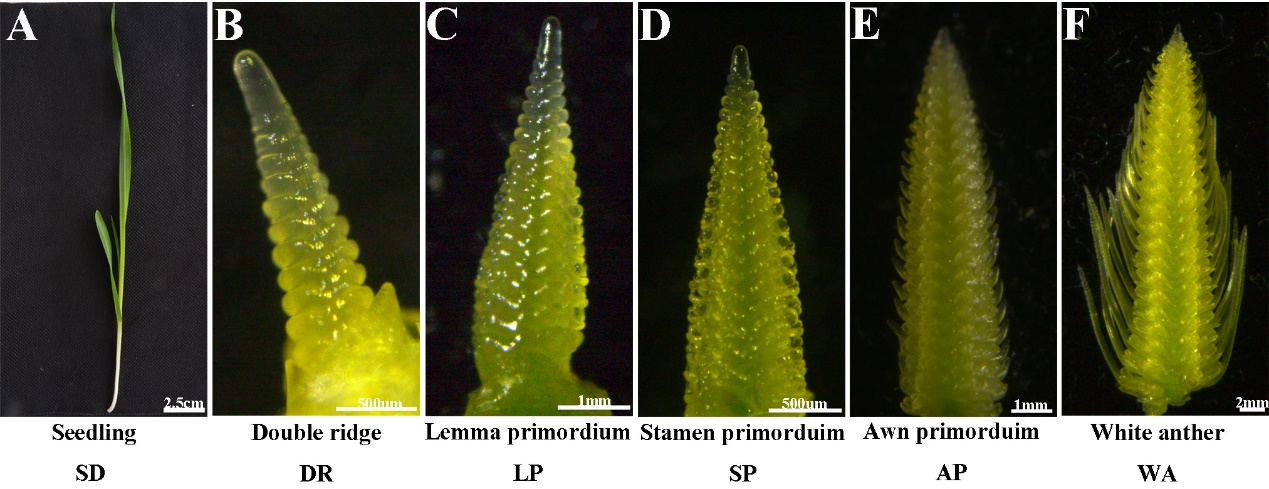


**Supplementary Fig. 5** Schematic representation of spike primordial development with stereoscope showing major barley spike initiation stages. SD: two-week-old seedling; DR: double ridge stage; LP: lemma primordium stage; SP: stamen primordium stage; AP: awn primordium stage; WA: white anther stage.
